# Clinical Decision Support Systems in Breast Cancer: A Systematic Review

**DOI:** 10.3390/cancers12020369

**Published:** 2020-02-06

**Authors:** Claudia Mazo, Cathriona Kearns, Catherine Mooney, William M. Gallagher

**Affiliations:** 1 UCD School of Computer Science, University College Dublin, Dublin 4, Ireland; claudia.mazovargas@ucd.ie (C.M.); catherine.mooney@ucd.ie (C.M.); 2 CeADAR: Centre for Applied Data Analytics Research, Dublin 4, Ireland; 3 OncoMark Limited, Dublin 4, Ireland; cathriona.kearns@ucd.ie; 4 UCD School of Biomolecular and Biomedical Science, UCD Conway Institute, University College Dublin, Dublin 4, Ireland

**Keywords:** breast cancer, clinical decision support system (CDSS), decision support system (DSS), systematic review, breast cancer treatment

## Abstract

Breast cancer is the most frequently diagnosed cancer in women, with more than 2.1 million new diagnoses worldwide every year. Personalised treatment is critical to optimising outcomes for patients with breast cancer. A major advance in medical practice is the incorporation of Clinical Decision Support Systems (CDSSs) to assist and support healthcare staff in clinical decision-making, thus improving the quality of decisions and overall patient care whilst minimising costs. The usage and availability of CDSSs in breast cancer care in healthcare settings is increasing. However, there may be differences in how particular CDSSs are developed, the information they include, the decisions they recommend, and how they are used in practice. This systematic review examines various CDSSs to determine their availability, intended use, medical characteristics, and expected outputs concerning breast cancer therapeutic decisions, an area that is known to have varying degrees of subjectivity in clinical practice. Utilising the methodology of Kitchenham and Charter, a systematic search of the literature was performed in Springer, Science Direct, Google Scholar, PubMed, ACM, IEEE, and Scopus. An overview of CDSS which supports decision-making in breast cancer treatment is provided along with a critical appraisal of their benefits, limitations, and opportunities for improvement.

## 1. Introduction

Breast cancer is one of the most common types of cancer in European countries and the second leading cause of cancer death. However, mortality rates are declining as a result of earlier diagnosis and improved therapies [1,2]. A breast cancer diagnosis impacts upon the health, lifestyle, work and family lives of affected individuals [3]. It carries with it not only a risk of severe morbidity and mortality, but side-effects of treatments that can leave physical and psycho-social complications that extend beyond treatment completion. An additional burden due to this disease can be economic hardship caused by loss of working hours and healthcare costs [4]. Therefore, breast cancer clinicians need accurate tools to help guide clinical decisions to improve patient prognosis, survival and quality of life whilst also reducing associated healthcare costs [4].

One of the opportunities and challenges of contemporary healthcare is the inclusion of digital tools in health education and service provision. Physicians, medical experts, and other healthcare staff, in collaboration with the patient, make decisions at different stages of the patient’s clinical journey, such as diagnosis, treatment, and surveillance. Precise, personalised and cutting-edge support tools are needed to ensure that the best decision is made [5,6]. Given the medical complexity of breast cancer, limited clinician time, and the need to make well-informed evidence-based decisions in a shared decision-making environment, the presence of an automated objective system to support decision-making has significant merit.

**Figure 1:**
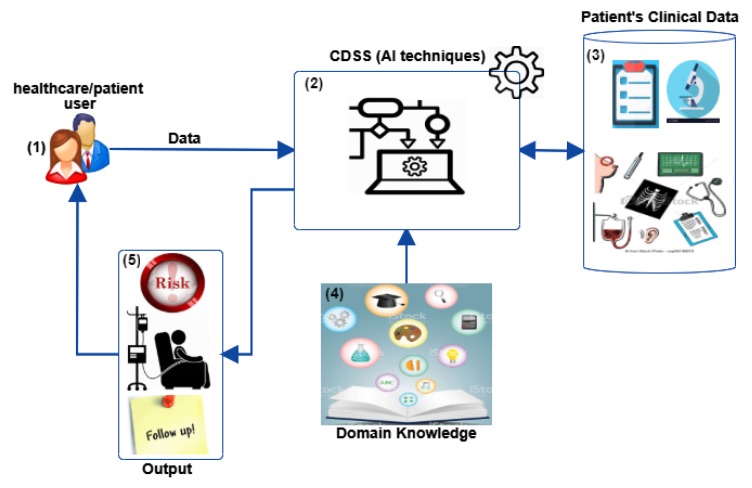
General diagram of a CDSS. (1) User (healthcare or patient) who provides information; (2) CDSS that uses Artificial Intelligence (AI) techniques to analyse data to help healthcare providers make decisions and improve patient care; (3) patient’s clinical data which may include several types of patient information such as personal details, medical tests, family history, environmental factors, amongst others; (4) domain knowledge is included in CDSS logic; (5) output of CDSS that could provide information about risk, treatment or follow-up.

A Decision Support System (DSS) is an information system that supports business, medical, educational or organisational decision-making activities. DSSs fuse human knowledge and technology to support and improve decision-making. A general diagram of a Clinical Decision Support System (CDSS) is presented in Figure 1. A CDSS has two principal action areas: (i) clinical decision-making and (ii) medical education [7]. Currently, CDSSs are used in many medical areas to improve healthcare system services [8], decision timing [8], and health-related quality of life for patients [8]; such systems can reduce both healthcare costs [8,9,10] and medical error rates [11,12,13]. CDSSs have potential benefits and drawbacks in implementation and use in clinical practice, specifically in oncology [14]. Coiera [15] defines the benefits of digital CDSSs as being three-fold: (i) patient safety, (ii) quality of care, and (iii) healthcare delivery efficiency. First, CDSSs can potentially improve patient safety by reducing adverse events, suggesting prescriptions tailored to the individual, and more efficient ordering of tests and medications [8,15]. Second, CDSSs may improve quality of care by reducing clinician’s workload, thereby making more time available for direct patient care [8,15], boosting the application of clinical pathways and guidelines by facilitating the use of up-to-date clinical evidence, and enhancing clinical documentation—essential for minimising errors and maximising patient satisfaction during decision-making [8,15]. Third, CDSSs can improve healthcare delivery efficiency, reducing costs by reducing test duplication, decreasing adverse events, and favouring less expensive but equally effective generic brands [8,15].

The area of precision oncology that supports the treatment of breast cancer has not only seen an increase in the availability of different treatment choices but also genomic tools to support the decision-making process [16]. CDSSs have been available for use in clinical oncology practice for over a decade [17]. However, awareness of CDSS types and their use in breast cancer treatment is limited in general. There has been some criticism around the utility of CDSS in personalised medicine decision-making of breast cancer, with these systems often viewed as aids better suited to support the ‘average’ patient’s requirements rather than personalised treatment for the individual patient [17].

This review aims to provide an overview of digital CDSS tools available to aid breast cancer treatment decisions. It adds to the existing literature by summarising trials of digital CDSSs implemented for breast cancer, critically appraising their properties and identifying their benefits, limitations and opportunities for improvement.

## 2. Method

The study of CDSSs for breast cancer treatment was conducted according to the methodology of Kitchenham and Charter [18] and was broken into three stages: (i) planning the review—related works and need for the review, and research question; (ii) conducting the review—data sources, and extracting data and synthesis, and (iii) results—what CDSSs are being used, how are they being used, what are the advantages and disadvantages of CDSS use, and what are the effects of CDSS use on patients with breast cancer.

### 2.1. Planning the Review

#### 2.1.1. Related Works and Needs for the Review

The literature which compares and contrasts available CDSSs for breast cancer treatment is very limited to the best of our knowledge. Initially, a total of nine reviews on general CDSSs were selected, using a systematic search explained in Section 2.2.1, to contextualise the study [8,19,20,21,22,23,24,25,26]. The focus of reviewing these papers was to assess and characterise CDSSs in a general context. This included evaluating clinical and economic outcomes, across a spectrum of diseases not specific to the treatment of breast cancer, which is a complex disease that involves combinations of systemic therapy. Indeed, multiple clinical, pathological and patient risk factors are evaluated to determine the appropriate treatment regimen for breast cancer patients. The use of CDSSs has, therefore, the potential to support both the clinician and patient in shared-decision-making of valid and applicable information that is both personalised to the individual and evidence-based regarding current recommendations. In recognition of the gap within the existing literature, we also conducted a systematic search of the electronic bibliographic databases from January 2008 to September 2019 for CDSSs specifically covering the subject of breast cancer treatment.

#### 2.1.2. Research Question

The research questions are: (i) to identify and critically appraise what CDSSs are being used in breast cancer treatment and their targeted outcomes; (ii) to evaluate, based on the literature, the advantages and disadvantages of using CDSSs in breast cancer treatment; (iii) to evaluate the effects on patients’ lives as a consequence of CDSS use.

### 2.2. Conducting the Review

#### 2.2.1. Data Sources

We conducted a systematic search of the literature in the following scientific and academic databases and search engines: Springer, Science Direct, Google Scholar, PubMed, ACM, IEEE, and Scopus. The searches were conducted in English and Spanish. Only CDSSs for breast cancer were selected; however, some DSSs and other types of CDSSs were added to provide general context. The following key terms were searched in the title, abstract or keywords of the published papers: “clinical decision support system”, “decision support system”, “breast cancer”, “systematic review”, “review”, “barriers to treatment”, and “prognosis”. Other key terms were used to refine and focus the search and irrelevant papers were excluded. We searched for studies reported between 1 January 2008 and 1 September 2019. A total of 1,227 papers were found at this stage before excluding irrelevant papers.

**Figure 2:**
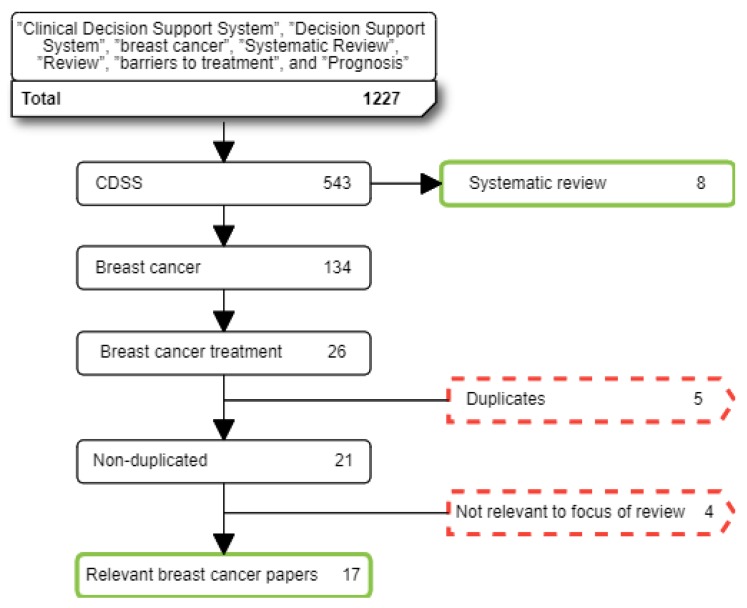
Flow diagram summarising the literature search, inclusion, and exclusion process. Red dotted squares correspond to excluded paper; green continuous squares correspond to selected papers.

The following seven exclusion criteria were applied to papers based on the purpose of our systematic review: (i) studies that do not use a CDSS or DSS in breast cancer; (ii) studies with limited/poor methods; (iii) studies with insufficient information on methods; (iv) studies of CDSS that focused on risk factors or screening for breast cancer—our focus was on treatment settings with patients who have breast cancer; (v) papers available only in the form of abstracts or PowerPoint presentations due to insufficient detail; (vi) papers that were not written in English or Spanish; and (vii) studies of CDSS not implemented in a clinical setting. A total of 17 papers were finally selected in this stage (see Figure 2). An additional eight systematic reviews were added to give support to the general context (see Figure 2).

#### 2.2.2. Extracting Data and Synthesis

In order to verify the quality of the selected studies, each study that met the inclusion criteria was abstracted by two reviewers independently and a questionnaire was completed for each paper. Each question was designed to elicit information about potential limitations in the quality of the study. The evaluation questions were: (i) Was the CDSS well described (what, how, who, where)?; (ii) Was the study population (i.e., number of patients, number of physicians) well described?; (iii) Did the authors specify how to access the CDSS?; (iv) Was the clinical question well described and covered (i.e., aim, output)? Answers that suggested quality limitations were evaluated as to whether they were of sufficient magnitude to reduce confidence in the results. Disagreements between reviewers during the process were resolved by consensus.

## 3. Results

The included studies were analysed to answer our three research questions. The first research question “to identify and critically appraise what CDSSs are being used in breast cancer treatment and their targeted outcomes” is covered in Section 3.1. There, we present an analysis of the available CDSSs for breast cancer treatment taking into account their target outcomes, input, method, results, main users, available format, access path, clinical question and dataset(s), among others. Table 1 summarises the most important characteristics and supporting evidence of the studies and help answer our research questions. The second research question “to evaluate, based on the literature, the advantages and disadvantages of using CDSSs in breast cancer treatment” is covered in Section 3.2. The third research question “to evaluate the effects on patients’ lives as a consequence of CDSS use” is covered in Section 3.3.

### 3.1. CDSSs for Treatment Decisions in Breast Cancer

CDSS tools for breast cancer treatment decisions make use of different methodologies—machine learning/artificial intelligence, knowledge representation, information visualisation, text mining, among others. Here, we present a selection of some of the most relevant CDSS tools to support decision-making for breast cancer treatment.

Adjuvant! Online was one of the original web-based tools used to assist clinical decision-making regarding the benefit for a particular breast cancer patient to undergo adjuvant treatment [27]. Mortality risk using this approach was based on SEER data for women aged 36–69 years and estimates of the efficacy of adjuvant therapy. This allowed for a discussion around competing mortality risks for older patients and those with comorbidities. Entering information on age and selected tumour characteristics—tumour size and grade, number of positive axillary nodes, and hormone receptor status—allowed for the prediction of the 10-year risk of relapse-free and overall survival [27]. Its use was validated in 4083 Canadian women [36]. However, validation studies of British [37] and Irish [38] women showed that it significantly over-estimated survival. Adjuvant! Online had several other limitations; for example, the relapse estimates include local-regional recurrence, as well as distant metastases so the overall survival estimate correlated better with predicted risk of distant recurrence when discussing adjuvant chemotherapy. Genomic assays such as Oncotype DX report risk of distant recurrence as the outcome used to decide whether chemotherapy is indicated. Locoregional relapses are more heavily influenced by the type and extent of locoregional therapy i.e., surgery and radiation. Adjuvant! Online did not incorporate HER2 status or percentage positivity for ER and PR. Practice changing data on the role of ovarian suppression, bisphosphonates, non-anthracycline-based anti-HER2 directed therapies, extended endocrine therapy and genomic assays, for example, were not included in the dataset. Additionally, Adjuvant! Online is not currently available.

PREDICT [28,29,39] is an online tool that helps patients and clinicians see how different treatments for early invasive breast cancer might improve survival rates after surgery. PREDICT uses patient age, tumour size, tumour grade, number of positive nodes, ER status, HER2 status, Ki67 status and mode of detection. PREDICT gives overall and breast cancer-specific survival for women treated for early breast cancer in the UK. The investigators used the Eastern Cancer Registration and Information Centre (ECRIC) dataset to develop this approach, which contained information from 5694 women who had surgery for invasive breast cancer in East Anglia from 1999 to 2003. An external dataset of 5468 patients from the West Midlands Cancer Intelligence Unit (WMCIU) was used for validation. This is emerging as a widely used tool by physicians due to its ease of use and access [29].

The ONCOassist breast adjuvant tool is based on the PREDICT algorithm [28,29] and is designed for oncology professionals. ONCOassist is a CE-marked CDSS available as a free smartphone or web app [30]. This tool gives prognostication and predicted treatment benefit to help clinicians and patients make informed decisions about treatment following breast cancer surgery. The tool provides survival estimates both with and without adjuvant therapy (hormone therapy, chemotherapy, and trastuzumab) for five and ten years following surgery. In this way, the specialists can better estimate breast cancer survival and quantify the benefits of hormone therapy, chemotherapy and trastuzumab. ONCOassist captures and provides the same information as PREDICT. The results are represented by graphics and text formats. ONCOassist also has applications in other tumour types, including colon and lung cancer. The smartphone or web app is personalised based on the user’s speciality and needs (specific tools, most relevant formulas, news feeds, etc.).

Oncotype IQ [31,40] brings together the Oncotype DX prognostic assays embedded within a breast cancer decision support algorithm. The oncologist enters the Risk Score (RS), patient age at surgery, tumour grade, and size and planned endocrine therapy. It does not consider co-morbidities, use of ovarian suppression, duration of endocrine therapy or nodal status. The algorithm recommends Hormonal Therapy (HT) alone for low RS, or chemotherapy followed by HT for high RS. Their dataset was composed of 643 patients, from UPMC Cancer Center, who had ER-positive, HER2 Neu-negative disease with zero to three positive nodes. Patients with treatment decisions made for chemotherapy without RS were also included. They used 21 genes for the recurrence score (16 cancer and five reference genes). However, users are required to register/subscribe to use this service.

DESIREE [32,41] is a web-based tool for the personalised, collaborative and multidisciplinary management of primary breast cancer by specialised Breast Units. DESIREE considers from diagnosis to treatment and follow-up. Patients’ cases are represented using a novel complex Digital Breast Cancer Patient (DBCP) model, which incorporates information about the patient’s clinical history and diagnostic and therapeutic procedures in cycles that may last for years. DESIREE DBCP includes medical imaging data from different modalities, biological and genetic data, novel diagnostic tests and biomarkers, risk factors, and clinical trials. The tool integrates data mining and visual analytics tools to exploit the multiple information available from retrospective cases and to be able to compare it with the current case. Their dataset is composed of data from 400 patients. However, their dataset is not publicly available and environmental or social aspects are not specified. It is important to highlight that this project is still in development and this tool is not free to access.

CancerMath [33,42] is composed of five online tools which predict the clinical outcome for individual cancer patients, as well as for accurately estimating the potential benefits of treatment with endocrine therapy and chemotherapy based on age, HER-2 status, tumour size, number of positive lymph nodes, tumour phenotype, and grade. These calculators are produced by the CancerMath.net group, a section of the Laboratory for Quantitative Medicine. These tools estimate therapy benefit with survival at 15 years. The results are presented in terms of breast cancer survival, treatment for each patient, probability of having positive nodes and risk of cancer in the nipple. CancerMath used the Surveillance, Epidemiology, and End Results Program (SEER) dataset composed of 779,999 records. The results can be represented in different ways—mortality curves, survival curves, bar graph, pie chart and pictograms—which are easy to use and can be understood both by experts and patients. Additionally, this tool is free to use under copyright law, with open access if you agree to all of the terms and conditions of use.

CTS5 Calculator [34] is a tool which predicts late distant recurrence for women diagnosed with ER-positive, primary breast cancer who are recurrence-free after five years of endocrine therapy. This calculator was developed by investigators from Queen Mary University, London and estimates therapy benefit, as determined by survival at 10 years. ATAC [43] and BIG1-98 [44] datasets were used to develop the CTS5 tool. The required inputs into this CDSS are tumour size, tumour grade, patient age, and the number of nodes involved. The results are presented in terms of CTS5 score, 10-year distant recurrence risk, and CTS5 risk group (low, intermediate and high) reported visually and in text format. Women are deemed to be at low risk of developing a late distant recurrence if their 5–10-year risk is less than 5%, intermediate between 5–10%, and women are deemed high risk for late distant recurrence if their 5–10-year risk is more than 10%. The low-risk group may not benefit from extended endocrine therapy and women who are in the high-risk group might see a benefit from extended endocrine therapy for the prevention of late distant recurrence. CTS5 is intended to be used by clinicians and results should be discussed with patients, including potential benefits and risks of extended endocrine therapy.

Residual Cancer Burden Calculator [35] is a prognostic tool for event-free survival and distant relapse-free survival. This calculator was developed by researchers at the MD Anderson Cancer Center in Houston. These investigators pooled data on over 5,000 patients from a dozen sites. Five input data types are required to use this tool, namely primary tumour bed area, percentage of cancer cellularity, percentage of cancer that is in situ disease, number of positive lymph nodes, and diameter of largest metastasis. The results are presented in terms of RCB and RCB Class which are classified patients as having minimal (RCB-I), moderate (RCB-II), or extensive (RCB-III) disease burden based on a continuous index.

Different predictive methods are used by the selected CDSS. Kaplan Meier analysis by Adjuvant! Online, cox regression models by PREDICT and ONCOassist, OncoType DX by Oncotype IQ, knowledge-based and image processing by DESIREE, and statistical algorithm by CancerMath, CTS5 Calculator and Residual Cancer Burden Calculator. Currently, statistical algorithms are used most frequently. Evaluating the overall performance of these CDSSs is challenging as they have different input features, provide a variety of predictions, and have been independently validated using different evaluation metrics.

Additionally, there are several other issues which we have identified. First, the low numbers of CDSSs available to support clinical decision-making in breast cancer treatment. Second, a number of the available CDSSs have been developed using demographic data from specific populations which have been shown not to generalise to other populations [45,46,47]. For instance, Adjuvant! Online used patients from the United States between the ages of 36 and 69 and PREDICT used patients from the United Kingdom between the ages of 40 to 70+ years. There are plenty of studies of age and demographic differences which compare aggressive cancers and lower survival rates [45,46,47], biological differences [48], rates of breast cancer [47], and breast cancer deaths [47]. Differential access to healthcare and the quality of healthcare available may explain the distinction between studies in different populations on breast cancer. However, research continues to investigate access to healthcare and the quality of healthcare influence on breast cancer treatment.

Third, none of the CDSSs to date incorporate all of the relevant variables. In some cases, clinicians must attempt to overlay studies relevant to a patient on top of datasets that have not included that information. For instance, most do not include ovarian suppression data for premenopausal women, genomic test results, or even ER or PR status, or the entry of co-morbidities.

Fourth, the area of breast medical oncology is a rapidly advancing field. CDSSs need to keep up with new data that emerges over time concerning breast cancer treatments, such as extended endocrine therapy, ovarian suppression particularly in under 35-year-olds, adjuvant bisphosphonates in postmenopausal women, and de-escalation of therapy in certain subgroups, such as HER2-positive patients with small tumours or patients who achieve pathological complete responses as more patients are treated in a neoadjuvant setting. None of the current CDSSs incorporates all of these features, which are very much part of what an oncologist currently discusses with patients relating therapy choices.

### 3.2. Advantages and Disadvantages of Using CDSSs

Oncologists use patient and tumour characteristics to make decisions regarding treatments for breast cancer. Patient factors include the patient’s age, co-morbidities and patient choice/wishes. Tumour factors include histological subtype, size, nodal status, grade, percent, ER, PR and HER2 expression and results of genomic tests, and staging investigations. However, the decision process may vary due to its subjectivity [49,50]. The incorporation of digital tools, such as CDSSs, using clinical outcome data can support the treatment decision process for breast cancer.

Potential benefits and disadvantages of using CDSSs were identified from reviews [15,51,52,53,54,55,56]. Advantages include: (i) saving time e.g., CDSSs provide immediate feedback to clinicians and patients, (ii) saving money, (iii) a better understanding of clinical situations for all users (clinicians, patients, health service administrators, among others), (iv) better use of resources e.g., CDSSs can help overcome problems due to inefficient coding of data, (v) medical education and training, (vi) suggesting alternative or additional treatment options, (vii) make new viewpoints, (viii) reduce variation in the quality of care, (ix) development and facilitation of inter-professional communication, (x) provide control, (xi) improve decisions, (xii) facilitate teamwork in medical staff, (xiii) facilitate clinical group decision-making, (xiv) provide new learning, (xv) ability to respond fast to unexpected situations, and (xvi) provide an audit trail and support research.

CDSSs also have some disadvantages [15,51,52,53,54,55,56], such as: (i) acceptance process—CDSSs can be perceived as a threat to clinical judgment by some physicians, there may be a fear of learning or getting out of their comfort zone, or fear of implementing new technology; (ii) promote over-reliance on software—physicians could think that CDSSs could limit their freedom to think; (iii) initial cost—e.g., maintenance, support, and training required; (iv) over-emphasis on decision-making—computerised decisions are focused on considering all aspects of a problem all the time, which may not be required in many of the situations. To overcome such barriers, it is important to adequately train the users to ensure effective and optimal use of CDSSs.

### 3.3. Effects of the CDSSs in the Population

Breast cancer is a heterogeneous disease in both its pathology and clinical pathway, making decision-making complex. Multiple factors that determine the choice of treatment for patients with breast cancer. Treatment decisions need to consider personal characteristics such as age, menopausal status and co-morbidities that the individual may have but also clinicopathological disease characteristics such as ER status, tumour grade, size, and number of involved axillary nodes all of which have to be balanced against the risk of recurrence or death with the potential benefit of the treatment on survival and impact on the patient’s quality of life. There is evidence that the use of decision-making aids may decrease patient anxiety and uncertainty regarding decision-making [57]. There is also growing evidence that supports excellent prognosis in subsets of breast cancer patients such as those classified as being node-negative or at low-risk of disease recurrence without the use of systemic therapy or treatment with individual treatments such as tamoxifen alone [58]. The complex heterogeneity of breast cancer, combined with a growing body of evidence of national and international guidelines on breast cancer treatments create a complex decision-making process for both clinicians and patients to make informed decision-making on the best adjuvant therapy for individual patients, highlighting the need for better ways of making treatment decisions in this area [59]. Shared-decision-making between patients and clinicians can be seriously compromised without effective mechanisms to aid understanding of benefits and risks for outcome probabilities. Research in clinical decision support has shown that their use can reduce costs, improve quality of care and reduce medical errors in clinical settings [60].

## 4. Discussion

In this study, we systematically reviewed the literature published between January 2008 and September 2019 on CDSS tools to determine their utility concerning assisting breast cancer treatment decisions. We considered papers that were written in both English and Spanish. Each CDSS is described, and an overview of features is given for comparative purposes. Our study shows the types of clinical and pathological inputs that inform each of the CDSS outputs.

In answer to our first research question to identify and critically appraise the use of CDSSs in breast cancer and their targeted outcomes, clear evidence is seen in studies on the effectiveness of CDSSs in healthcare in general [20], and specifically, cardiovascular diseases [21], pulmonary disease [61], chronic wounds [62], drug prescribing [63] and prostate cancer treatment [64], among others. This type of evidence is also needed for CDSS tools specific to breast cancer treatment decision-making. Our systematic review returned 17 papers on CDSSs which support decision-making in breast cancer treatment. While the number of CDSSs developed specifically for breast cancer is limited, our study demonstrated that there are different types of treatment decisions that can be supported by CDSSs regarding chemotherapy, surgery, hormonotherapy, radiotherapy, and follow-up.

As regards our second research question, namely to understand the advantages and disadvantages of CDSS use, we found that current CDSSs could potentially improve services within the healthcare system, time to decision, and health-related quality of life for patients; and reduce both healthcare costs and medical error rates [8,12]. However, as with any healthcare innovation, CDSSs should be rigorously assessed before dissemination into clinical practice, i.e., corroborate that indeed it improves clinical care or patient outcomes. Additionally, some controlled trials are warranted [65]. Medical errors are both costly and harmful [66]. A mistake in breast cancer treatment decisions can have catastrophic implications for health-related quality of life and outcome. A review study on CDSS between 1990 and 2007 found that CDSSs could play a significant role in boosting healthcare systems and physicians’ performance. In general, the findings of the present study are aligned with the conclusions presented in [67].

The CDSSs identified in the study focus principally on the prediction of survival that informs treatment benefit. Most of the tools include clinical and pathological risk factors. However, there is variation in the risk factors included, for example, patient age, tumour size, tumour grade, number of positive nodes, ER status, HER2 status, Ki67 status, imaging data, and among others [28,30,32,41]. The patient cohort that the CDSSs are developed with the need to be representative of the population who will use the CDSS. Adjuvant! Online, for example, was developed on a Canadian population and then shown to significantly over-estimate survival in a UK population [37]. Our study shows that a number of breast cancer CDSSs are available online [17,28,30,31,33,41]. However, not all are free to access, with users having to pay to use some tools or to use additional features.

It is important to highlight how CDSSs could change the effect on decision outcomes, especially for treatment decisions for breast cancer patients. A follow-up before-after study on the use of one CDSS, OncoDoc [68], which looked at 127 treatment cases found that the use of OncoDoc increased compliance with clinical practice guidelines from 61% to 77%. The authors concluded that initial and final therapeutic decisions were not identical in 31% of the cases (39/127). Among the 16 cases in which physicians’ initial decision was not compliant at all with OncoDoc’s recommendations, physicians changed their mind to comply with OncoDoc in 62% of the cases (10/16). However, in six cases, although they modified their decision, they remained non-compliant with OncoDoc.

This review aimed to identify, compare and contrast the CDSS tools available to aid breast cancer treatment decisions. By doing so, we hope to highlight the value and clinical practice limitations of the tools that are currently available and inform areas for improvement for future developments in this area. We chose breast cancer treatment as an area for consideration due to the complex heterogeneity of the disease and subsequent difficulty in making treatment decisions. Thus, CDSSs can significantly contribute to this area.

## 5. Conclusions and Future Work

CDSSs have potential benefits in implementation and use in clinical practice. CDSSs are effective in improving screening for different diseases risk factors and clinician practices for preventive care services, clinical tests, treatments, improving decision-making about which interventions to implement, and follow-up [67].

There are not many CDSSs based on breast cancer treatment decisions on the market. However, to date, there has not been an established/reported global standard or organisation to provide supervision on either the development or application of CDSSs in healthcare systems [69]. We recommend the provision of a guide or quality control system to verify the evaluation of CDSSs in terms of development and application.

CDSS tools for breast cancer treatment decisions use different inputs, for example, medical imaging data, biological and genomic data, diagnostic tests and biomarkers, risk factors, social aspects, clinical trials, among others. It could be very interesting to include environmental data and lifestyle risk factors to provide more accurate and personalised treatment decision for breast cancer patients. The differences in inclusion/exclusion criteria for CDSSs can impact on the predicted outcomes. As the availability of new genomic tests and drugs come onto the market, CDSSs need to be able to adapt accordingly. Indeed, it is critical to consider other relevant variables such as ovarian suppression for premenopausal women, genomic test results, ER or PR status, co-morbidities, the result of extended endocrine therapy, ovarian suppression particularly in <35-year-olds, adjuvant bisphosphonates in postmenopausal women, de-escalation of therapy in some subgroups such as HER2 positive patients with small tumours or patients who achieve pathological complete responses. This point should be considered as part of a CDSS flexible to emergent data.

Our literature search screened more than 1000 articles to identify potentially relevant studies. The study provides an overview of differences in features, user availability, decision routes and outcomes for breast cancer CDSS. Our study focused on exploring CDSSs for assisting breast cancer treatment decisions as opposed to breast cancer risk and provides preliminary evidence for the current status of CDSSs in breast cancer treatment decision-making. 

## Figures and Tables

**Table 1:** Overview of reviewed Clinical Decision Support Systems *.

CDSS	Users	Sample	Clinical Question	Clinical & Pathological Input	Method	Access	URL
Adjuvant! Online [27]	HC	631	Risk of recurrence and death at 10 years; Estimated benefits of adjuvant endocrine and chemotherapy	Surveillance, epidemiology, SEER, and efficacy of adjuvant therapy	Kaplan Meier analysis	Not available	www.adjuvantonline.com
PREDICT [28,29]	HC/P	5694	Prediction of survival at 5, 10 and 15 years with different treatment options	Age, tumour size and grade, PN, ER, HER2, Ki67 and mode of detection	Cox regression models	Online	https://breast.predict.nhs.uk/
ONCOassist [30]	HC	5694	What is the most appropriate treatment following breast cancer surgery? Prediction of survival at 5, 10 and 15 years	Patient age, tumour size, tumour grade, number of positive nodes, ER status, HER2 status, Ki67 status and mode of detection	PREDICT method (Multivariable Cox regression models)	Online /smartphone app	https://oncoassist.com/
Oncotype IQ [31]	HC	643	Do I have aggressive disease? Do I need radiation? Do I need surgery? Do I need chemo? What stage is my cancer?	HER2-, ER+, PN	OncoType DX	Online	https://online.genomichealth.com
DESIREE [32]	HC/P	400	What are the available therapy options?	Medical imaging, biological and genetic data, diagnostic and biomarkers, risk factors, environmental or social aspects, clinical trials	Image processing, ontology and datamining	Online	http://www.desiree-project.eu/
CancerMath [33]	HC/P	779,999	Prediction of survival at 15 years with different treatment options	SEER, HER-2, tumour size, nodal, tumour phenotype, and grade	Statistical algorithms	Online	http://www.lifemath.net/cancer/
CTS5 Calculator [34]	HC/P	4000+	Prediction of survival at 10 years with different treatment options	Tumour size, tumour grade, patient age, and number of nodes	Statistical algorithms	Online	www.cts5-calculator.com/
Residual Cancer Burden Calculator [35]	HC/P	5000+	Prognostic for event-free and distant relapse-free survival	Primary tumour bed area, percentage of cancer cellularity, percentage of cancer that is in situ disease, lymph nodes, number of positive lymph nodes, and diameter of largest metastasis	Statistical algorithms	Online	http://www3.mdanderson.org/app/medcalc/index.cfm?pagename=jsconvert3

* BC: breast cancer, P: patient-oriented interface, HC: Healthcare professionals’ interface, PN: Positive Nodes, HRS: hormone receptors status, CDSS: Clinical Decision Support System.

## Abbreviations

The following abbreviations are used in this manuscript:

CDSS	Clinical Decision Support System
DSS	Decision Support Systems
HT	Hormonal Therapy
RS	Risk Score
DBCP	Digital Breast Cancer Patient
WMCIU	West Midlands Cancer Intelligence Unit
SEER	Surveillance, Epidemiology, and End Results Program
EI	Enterprise Ireland
IRC	Irish Research Council
SFI	Science Foundation Ireland
